# The effect of the medicine administration route on health-related quality of life: Results from a time trade-off survey in patients with bipolar disorder or schizophrenia in 2 Nordic countries

**DOI:** 10.1186/s12888-016-0930-6

**Published:** 2016-07-16

**Authors:** Tine Rikke Jørgensen, Charlotte Emborg, Karianne Dahlen, Mette Bøgelund, Andreas Carlborg

**Affiliations:** Medivir AB, Blasieholmsgatan 2, 111 48, Stockholm, Sweden; OPUS Klinikken, Risskov, Denmark; Incentive, Holte, Denmark; Department of Clinical Neuroscience, Centre for Psychiatric Research and Education, Karolinska Institutet, Stockholm, Sweden

## Abstract

**Background:**

Agitation episodes are common among patients with schizophrenia or bipolar disorder. Oral and intramuscular administration methods are commonly used in pharmacological treatment of acute agitation. Recently, an innovative inhalation product with loxapine(Adasuve®)has become available for treatment of acute agitation episodes associated with bipolar disorder or schizophrenia. The objective for the present study was to investigate the impact of the pharmacological treatment’s administration methods on the health-related quality of life (HRQoL) in patients with bipolar disorder or schizophrenia in Denmark and Sweden using a time trade-off (TTO) approach.

**Methods:**

The TTO methodology was used to examine the HRQoL impact of administration method of pharmacological treatment of acute agitation. Data were collected via an internet-based survey, using an existing panel of respondents with schizophrenia or bipolar disorder.

**Results:**

Respondents considered living with schizophrenia/ bipolar disorder, having one yearly agitation episode treated with inhaler better than living with the same conditions and receiving treatment with tablet or injection. The utility value was 0.762 for inhalable treatment, 0.707 for injection and 0.734 for tablet treatment.

**Conclusions:**

Patients’ preference for treatment delivery options showed that inhalation was associated with a significant utility gain when compared to injection or tablets. Inhalable loxapine may be a new tool for control of agitation episodes for strengthening the patient provider alliance when taking patient’s preference for delivery method into consideration.

## Background

Agitation episodes are common among patients with schizophrenia or bipolar disorder [1, 2]. Agitation is a state where patients experience extreme alertness accompanied by restlessness and unorganized psychomotor activity. Inadequate or late control of acute episodes of agitation could drive escalation of symptoms to violent or aggressive behaviors and might have a significant impact on resource use and on patients’ lives [2]. Treatment of moderate or severe acute agitation is normally managed in hospital setting and includes both non-pharmacological and pharmacological interventions. Currently, there is a very strong focus in the Nordic countries on avoiding coercive treatment and restraints in the psychiatric wards- Initially, non-pharmacological methods of behaviour control, such as a verbal intervention or de-escalation techniques, may be helpful to manage agitated patients. When initial non-pharmacological methods fail to calm the patient, seclusion and/or physical restraint might be appropriate, or there may be a need to initiate pharmacological interventions. The available medical treatments for agitation include first- and second-generation antipsychotics and benzodiazepines, and three routes of administration are possible: Oral/oral fast-dissolving tablets, intramuscular, or intravenous [3]. Intravenous administration of antipsychotic drugs offers a rapid onset of action, but it is often impractical unless intravenous access is already established. As a result, oral and intramuscular administrations are more commonly used, but these routes entail a notably delayed onset of action. A non-coercive, collaborative approach to manage agitation is recommended, and oral antipsychotics are preferred in these collaborative interventions [4, 5]. Oral formulations may, however, sometimes be inappropriate due to the slow onset of action, which leaves a significant period during which patients, staff and property remain vulnerable to the deleterious behaviors associated with agitation [6]. The use of intramuscular injections may be limited by several barriers: Mental and physical trauma to the patient, risk of compromising the patient-physician relationship, exposure to contaminated needles and negative effects on long-term compliance [6]. It has been demonstrated that the subjective feeling after the first medication is essential for the acceptance of medication and the further course of the episode [7].

According to ‘The Expert Consensus Guidelines for Treatment of Behavioral Emergencies’, speed of onset, control of aggressive behavior, patient preference, long-term physician-patient relationship and medication reliability of delivery are identified as attributes for an anti-agitation pharmacological treatment, of which speed of onset is considered as most important by the expert panel [6].

The route of administration is known to have great impact on the time of onset and the elimination time of the drug. Recently, a new product with inhalation as delivery method has become available for treatment of acute agitation episodes associated with bipolar disorder or schizophrenia. Inhalable loxapine (Adasuve®) utilizes an antipsychotic used in psychiatry for 30 years with a well-established efficacy and safety profile [8]. Adasuve® is a single-dose inhaler that dispenses a low dose of loxapine (9.1 mg) directly into the lung tissue for rapid absorption with a mean time of maximum plasma concentration (T_max_) of 2 min, hence fast relief for patients during an agitation episode [8–11]. The drug-delivery system is extensively described elsewhere [12, 13].

In Denmark and Sweden, the standard medical treatments of agitation episodes include antipsychotics administered per oral/oral or by intramuscular injections. The efficacy of these is assumed to be comparable [14–16]. It is known from other therapy areas (e.g., oncology and diabetes) that patients prefer to receive the medication in the form of tablets or an inhaled preparation rather than as injections [17–19]. Oral or inhaled medications are also associated with heightened levels of patient satisfaction with their treatment [17, 20]. In Denmark the national Council for the Use of Expensive Hospital Medicines (RADS) guidelines and in Sweden the local “Wise Drug Formulary” guidelines are used with the aim of choosing the most suitable drug from an economical perspective, with the flexibility of choosing more expensive drugs if there is appropriate arguments in the patients’ files. In the current study, the time trade-off (TTO) methodology was used to examine the impact of administration method of pharmacological treatment of acute agitation on the health-related quality of life (HRQoL). The TTO method is recognized by the Health Technology Assessment agencies for direct health-state valuation and is used to calculate quality-adjusted life years (QALYs), and for cost-effectiveness calculations of treatments. The objective for the present study was to investigate the impact of the administration method, in connection with medical treatment of acute agitation, on health related quality of life (HRQoL) in patients with bipolar disorder or schizophrenia in Denmark and Sweden using a TTO approach.

## Methods

### Study design

The TTO method is a tool for eliciting utility values by asking respondents to ‘trade off’ a part of their remaining lifespan to live in an improved health state. The TTO method is described extensively elsewhere [21–29]. The time horizon used in the questionnaire was based on each respondent’s life expectancy. This was obtained by combining information on the country, age and sex of the respondents at the time of study and the most recent life tables from the World Health Organization [30]. Respondents were asked questions repeatedly, varying only the remaining years in full health in order to identify an interval around the point of indifference between the two hypothetical health states (where both options are equally acceptable). This procedure followed a standard bisection methodology, using a starting point of utility 0.6 and from there narrowing down the respondents’ interval around the point of indifference by presenting them with 4–5 trade-offs. The distribution tails were given particular attention. If respondents chose to trade a very high proportion of their remaining lifespan or chose not to trade any lifetime at all, their reasons for choosing so were carefully screened.

### Definition of health states

Health states were defined on the basis of patient experiences derived from focus groups, from applicable product summaries (SmPCs) from the European Medicines Agency and by consulting a Danish medical expert. Three health states were developed to elicit utility values of 3 treatment methods for agitation episodes (Table 1). The health states were described as involving ‘Injection’ , ‘Tablets’ and ‘Inhaler’. All health states were hypothetical, and the respondents were asked to imagine that they lived with schizophrenia or bipolar disorder and that they had one yearly agitation episode (’schizophrenia’ or’bipolar disorder’ was displayed, depending on the respondent’s condition). The only difference between the three health states was the type of treatment of the agitation episode. No brand names were presented in the questionnaire. For the health state including tablet or injection as treatment, olanzapine was chosen as a proxy, and inhalable loxapine was used as a proxy for the health state including inhalation as treatment of a yearly agitation episode. The respective T_max_ was used as the time to onset of efficacy of inhalation and injection treatments. Intramuscular injection with olanzapine has a T_max_ of 15–45 min, and in the questionnaire the time to efficacy was set to 30 min [31]. The time to efficacy for inhalation was set to 2 min (T_max_ for inhalable loxapine) [11]. The tablet formulation of olanzapine has a T_max_ of 3–6 h [31], but time to onset for oral formulations is reported as being earlier in the literature [32]. As a consequence, and to make the health states easier to compare and understand for patients, the time to effect for tablets was described as 1 h.

**Table 1:** Health state description for the TTO

Health State	Description
Injection	You say yes to medication and receive the medication as an injection. It takes approximately 30 min for the medication to work. During the period until the medication starts to work you are still exasperated, aggressive, restless, agitated or anxious. For approximately 1 day after you have taken the medication, in 1 out of 3 cases you risk feeling sluggish, feeling tired, having the bubble feeling or feeling groggy.
Inhalation	You say yes to the medication and receive the medication via an inhaler, which you use by exhaling, placing the inhaler in your mouth and inhaling, whereby you receive the medication. It takes approximately 2 min for the medication to work. During the period until the medication starts to work you are still exasperated, aggressive, restless, agitated or anxious. For approximately 7 h after you have taken the medication, in 1 out of 10 cases you risk feeling sluggish, feeling tired, having the bubble feeling or feeling groggy.
Tablet	You say yes to the medication and receive it in tablet form, which you swallow with a glass of water. It takes approximately 1 h for the medication to work. During the period until the medication starts to work you are still exasperated, agitated or anxious. For approximately 1 day after you have taken the medication, in 1 out of 3 cases you risk feeling sluggish, feeling tired, having the bubble feeling or feeling groggy.

Three health states (‘Injection’ , ‘Tablets’ and ‘Inhaler’) were developed for eliciting utility values of 3 treatment methods for agitation episodes. All health states included living with schizophrenia or bipolar disorder and having one yearly agitation episode. It was assumed that patients knew what it was like to live with their own condition (schizophrenia or bipolar disorder), thus the questionnaire did not describe that. TTO = Time trade-off

Only adverse reactions stated as very common and severe according to the respective SmPC were considered as relevant for the TTO methodology. As this study concerned treatment of acute agitation only, sedation was the only adverse reaction evaluated as applicable for the TTO methodology. Clinical studies have shown that approximately 30 % of patients receiving olanzapine administered by oral or intramuscular route will experience sedation as measured on the Agitation-Calmness Evaluation Scale (ACES = 7, 8 or 9) [31]. This adverse reaction may be experienced for at least 24 h after the treatment [33]. The half-life (T_1/2_) of olanzapine is 33 h, and at this time it is expected that the risk of sedation is no longer present. Yet it is not known from clinical studies if sedation continues after 24 h. Approximately 10 % of patients receiving inhalable loxapine will experience sedation [10]. In the health states describing tablet or injection as treatment for agitation, the respondents received the information that 1/3 will experience sedation for 24 h. The half-life of inhalable loxapine (Adasuve®) is 6–8 h, and no information is available on whether patients experience sedation beyond this point. In the questionnaire, it was stated that 1/10 of the patients will experience sedation for 7 h when treated with inhalation (Table 1).

### Questionnaire design

Focus group interviews with 11 patients with bipolar disorder and 3 patients with schizophrenia, together with their primary caregiver were conducted as a preparation for the current survey. The focus group discussions gave insights into how patients experience an agitation episode as well as their experiences with adverse reactions of the treatments. In the focus group, agitation was described by the patients as feeling like restlessness, anger, anxiety or impatience, hence a list of these words was used to describe the agitation in the questionnaire. The objective of including caregivers was to gain insight into the caregivers’ experience of supporting patients living with acute agitation and to discover the treatment characteristics most valued by patients and their caregivers.

During the discussions, patients mentioned that sedation in relation to medication for agitation felt like grogginess or a hangover effect. The medical expert consulted prior to conducting the current survey stated that patients describe a ‘bubble feeling’. In order to use patient terminology in the questionnaire, sedation was described as feeling ‘sluggish, tired, having the bubble feeling or feeling groggy’. The focus groups provided background insight tat informed for development of the survey questionnaire.

### Survey description

Data were collected via an internet-based survey, using an existing email panel of respondents who had previously agreed to participate in internet-based surveys. The respondents were anonymous and no sensitive information such as names or addresses were known to the authors. Inclusion criteria were consent to participate in the survey, a self-reported diagnosis of either schizophrenia or bipolar disorder and age above 18 years. The survey was conducted in Denmark and Sweden from 30 October 2014 to 12 December 2014 and in Danish and Swedish, respectively. The respondents received a remuneration (1 €) for participating in the survey. The questionnaire was programmed in a commercial survey software package, SurveyXact®. To improve answer quality and prevent unconsidered responses, a delay of 10 s was introduced to pages with a large amount of text. The functionality of the questionnaire was tested in a pilot study of 12 respondents.

### Exclusion criteria

Respondents were excluded if they refused to trade on ethical or religious grounds or if they stated they did not understand the question. On the other hand, if respondents believed the health state was manageable or if they expressed a desire to live as long as possible due to obligations (such as being a caregiver), their answers were kept in the further analyses. A test question was included to check the respondents’ understanding of the TTO concept. In this question, the respondents had to choose between 1) full health and a longer remaining lifespan and 2) a less than full health and a reduced lifetime. If respondents chose the second option, they were excluded from the TTO analysis.

### Statistical analysis

Statistical analyses were performed in SAS® version 9.4 statistical software. Based on patients’ trade-offs, a utility value was assigned to each of the three health states for all respondents. This value was calculated as the midpoint of the highest and the lowest utility values that the respondents stated for a specific health state. For example, if respondents answered that they had a utility higher than 0.80 and lower than 0.85, their utility was estimated to be 0.825. The average utility value was calculated for each health state, and subsequently the difference in utility between the different health states was calculated. The response distribution was non-normal, so we used non-parametric bootstrapping to simulate standard errors and confidence intervals (CIs) for the mean TTO values. When using this method, the parameter’s distribution is estimated by repeatedly resampling the original data set with replacements [34–36]. For the present study 10,000 iterations were used.

## Results

### Demographics

**Fig. 1:**
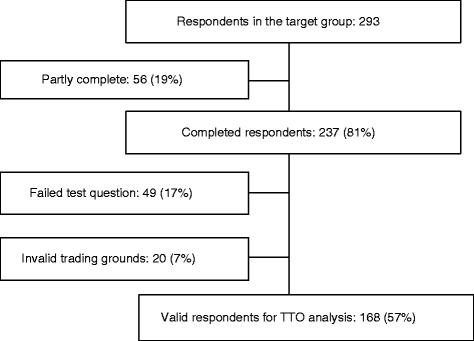
Patient consort flow diagram. TTO: Time Trade-Off

The number of eligible participants was 293 (176 with bipolar disorder and 61 with schizophrenia), of which 56 (19 %) did not complete the questionnaire, 49 (17 %) failed the TTO test question, and 20 (7 %) were excluded from analysis as they traded too much or too little on invalid grounds (such as religious or ethical grounds, or if they did not understand the questions) (Fig. 1). Therefore, 168 (57 %) respondents were included in the final analysis. The demographic characteristics of the final sample of respondents are summarized in Table 2.

**Table 2:** Demographic characteristic of the final sample

	All	Schizophrenia	Bipolar disorder
*N*	168	41	127
Male (%)	33	51	27
Female (%)	67	49	73
Average age (years)	40	34	42
Average age at diagnosis (years)	29	22	32
Have been hospitalized (%)	64	85	57
Have been hospitalized in the last year (%)	28	41	24

In total, there was a majority of female respondents (67 %); however, respondents with schizophrenia were more equally distributed with 51 % female respondents. The average age of the respondents was 40 years, and average age when diagnosed with their condition was 29 years. More respondents with schizophrenia had been hospitalized, of which 41 % were hospitalized within the last year. Among the respondents with bipolar disorder, 24 % had been hospitalized within the last year. Ninety per cent of the respondents had experienced an agitation episode. When patients themselves asked for medication during an agitation episode, most patients (85 %) asked for tablets. Among those patients who were given medication against their will during an agitation episode, 52 % of patients received injections and 29 % received tablets. Nineteen per cent received both injections and tablets. Eighty-one per cent of patients have experienced sedation when receiving medical treatment.

### TTO results

**Fig. 2:**
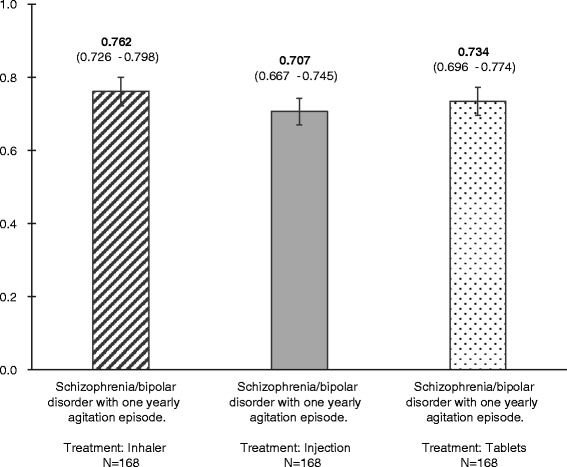
QALY value for the different health states. Respondents considered living with schizophrenia/bipolar disorder and having one yearly agitation episode treated with inhaler better than living with the same conditions and receiving treatment with tablets or injection. The utility value was 0.762 for inhalable treatment, 0.707 for injection and 0.734 for tablet treatment. QALY: Quality-adjusted life years

**Fig. 3:**
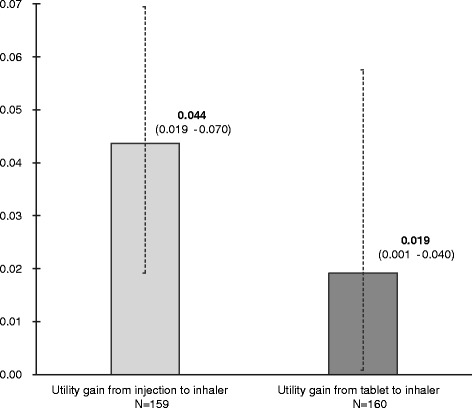
QALY gain associated with change in treatment of 1 yearly agitation episode. The utility differences between injection and inhaler treatment and between tablets and inhaler are significantly different at a 95 % CI as denoted by the error bars. QALY: Quality-adjusted life years

Figure 2 shows the utility of living with schizophrenia/bipolar disorder and having one yearly agitation episode for the 3 health states explored. Respondents considered living with schizophrenia/bipolar disorder, and having one yearly agitation episode treated with inhaler better than living with the same conditions and receiving treatment with tablets or injection. The utility value was 0.762 for inhalable treatment, 0.707 for injection and 0.734 for tablet treatment (Fig. 2). The differences in utility between the treatments are seen in Fig. 3. The utility difference between the injection and the inhaler treatment is 0.044, and the difference in utility between the tablet and the inhaler treatment was 0.019. The utility difference between injection and inhaler treatment and between tablets and inhaler are significantly different at a 95 % CI as denoted by the error bars in Fig. 3. This shows that patients gain a higher utility by receiving inhalable treatment compared with using injections or tablets.

## Discussion

This is the first study measuring patients’ self-reported utilities associated with different routes of medicine administration for control of agitation episodes using the TTO method. Agitation episodes are common among patients with schizophrenia or bipolar disorder; 90 % of the respondents in this survey had experienced at least 1 agitation episode. The majority of the respondents in this study had received intramuscular injection against their will; 52 % had received injection, and 19 % had received tablet and injection concomitantly against their will. A desirable goal of using medication for agitation is to calm the patient without inducing sleep, as it allows a patient-provider alliance in the creation of a crisis management plan. A recent consensus statement of the psychopharmacology workgroup of the American Association for Emergency Psychiatry recommends that patients should be involved, if possible, in both the selection of the type and the route of administration of any medication [3]. Inhalation offers an alternative route of delivery for pharmacological treatment of agitation episodes associated with bipolar disorder or schizophrenia. The absorption site and the lack of first-pass metabolism make the inhaled form of medication the one to offer the fastest onset of action, but it also requires some cooperation from the patient. In the current study, patients reported inhalation as the preferred administration method, as it was traded as the most valuable treatment, indicated by the highest QALY (Fig. 2). This indicates that patients might be willing and cooperative to receive treatment delivered by inhalation. Respondents traded injection as the least valuable route of administration, indicated by the lowest QALY (Fig. 2). Similar findings were found by Atkinson et al., who demonstrated significant differences in satisfaction levels by route of medication administration (oral, injectable, inhalable) across different diseases. Injectable was the least preferred route of administration regardless of disease [20].

As mentioned, this is the first study to elicit stated preferences for the administration method of medication for acute agitation in the form of health state preference value. These values, taking the form of a single index on a zero-to-one scale representing death and perfect health, respectively, may be used as preference weights in future economic evaluations. A UK study, including stable outpatients with schizophrenia and no side effects of treatment, obtained a utility value of 0.919, slightly higher than in the current study [37]. This is not surprising, as the current study included only 1 yearly agitation episode. In the UK study, the side effect of treatment lowered the utility value to 0.769, and extrapyramidal symptoms impaired the utility value to 0.722 [37]. In the current study, the utility value was 0.762 for inhalable treatment and 0.707 for injection, which supports the theory that inhalable treatment for agitation episodes would increase health utility in patients with bipolar disorder or schizophrenia compared with current treatments. The cost of inhalable treatment for agitation may exceed the cost of treatment with tablets; however, the preferences for inhaled administration may be sufficiently strong among potential users of inhalable treatment to justify the additional cost. Poor management of agitation among psychiatric patients can result in an inappropriate use of coercive measures and/or escalation to patient violence, both of which present a significant burden to patients and caregivers as well as a substantial economic burden to the healthcare system [2, 38]. This burden could be substantially lowered by improved clinical management of agitated patients, including the use of appropriate and rapid interventions to avoid escalation to violence and aggression.

The TTO instrument is relatively easy to use, which makes it more appropriate to use in the current study than the widely used standard gamble (SG) instrument as SG can be difficult for respondents to understand [28]. To use an age-dependent life-expectancy adaption of the TTO questions is to increase the relevance compared with the fixed 10- or 30-year trade-offs often used in other studies. The repetitive nature of the TTO questions might lead to fatigue among respondents. To avoid this, respondents evaluated a limited number of health states and were only given 4–5 iterative questions per health state plus two follow-up questions to clarify extreme answers (people who traded a lot or almost nothing). Because the survey was web-based, it could be argued that it is biased towards literate respondents with internet access and IT competences. The literacy rate and IT competences are generally very high in Sweden and Denmark, although it is possibly somewhat lower in the target group of this survey. The dropout rate of 19 % may seem high; however, this is typical of web-based surveys used in health-economic assessments [3–41]. The percentage of patients who failed test questions or were excluded on invalid trading grounds is also within limits of comparable studies [42, 43].

A limitation of this study is the generalizability. Since the study was conducted among a limited number of patients in Denmark and Sweden, the results may not be transferable to all patients in these countries or to patients in other countries. We do, however, assume that the results would reflect a preference observed in a western health care context.

## Conclusions

Agitation requires clinical intervention when the level of hostility, excitement, uncooperativeness or lack of impulse control is such that the potential exists for harm to self or others. Currently tablets and injections are used to control agitation episodes. Recently, a new rapidly acting inhalation form of loxapine for acute agitation became available for patients. This survey of patients’ preference for treatment delivery options showed that patients preferred inhalation as the delivery method. As a result, inhalation was associated with a significant utility gain when compared with injection or tablets. Inhalable loxapine might be a new tool for control of agitation episodes that can be used to strengthen the patient-provider alliance.

## Abbreviations

ACES, agitation calmness evaluation scale; CI, confidence intervals; HRQoL, health related quality of life; QALY, quality adjusted life years; RADS, Rådet for Anvendelse af Dyr Sygehusmedicin; SG, standard gamble; SmPC, summary of product characteristics; T_½_, half life; T_max_, mean time of maximum plasma concentration; TTO, time trade off

## References

[1] Alderfer BS, Allen MH (2003). Treatment of agitation in bipolar disorder across the life cycle. J Clin Psychiatry.

[2] Hankin CS, Bronstone A, Koran LM (2011). Agitation in the inpatient psychiatric setting: a review of clinical presentation, burden, and treatment. J Psychiatr Pract.

[3] Wilson MP, Pepper D, Currier GW, Holloman GH, Feifel D (2012). The psychopharmacology of agitation: consensus statement of the American Association of Emergency Psychiatry, project BETA psychopharmacology workgroup. West J Emerg Med.

[4] Marder SR (2006). A review of agitation in mental illness: treatment guidelines and current therapies. J Clin Psychiatry.

[5] Zeller SL, Wilson MP (2011). Acute treatment of agitation in schizophrenia. Drug Discov Today.

[6] Allen MH, Currier GW, Hughes DH, Reyes-Harde M, Docherty JP, Expert Consensus Panel for Behavioral Emergencies. The Expert Consensus Guideline Series. Treatment of behavioral emergencies. Postgrad.Med. 2001;(Spec No):1–88; quiz 89–90. 11500996

[7] Van Putten T, May PR, Marder SR, Wittmann LA (1981). Subjective response to antipsychotic drugs. Arch Gen Psychiatry.

[8] Kwentus J, Riesenberg RA, Marandi M, Manning RA, Allen MH, Fishman RS (2012). Rapid acute treatment of agitation in patients with bipolar I disorder: a multicenter, randomized, placebo-controlled clinical trial with inhaled loxapine. Bipolar Disord.

[9] Allen MH, Feifel D, Lesem MD, Zimbroff DL, Ross R, Munzar P (2011). Efficacy and safety of loxapine for inhalation in the treatment of agitation in patients with schizophrenia: a randomized, double-blind, placebo-controlled trial. J Clin Psychiatry.

[10] Lesem MD, Tran-Johnson TK, Riesenberg RA, Feifel D, Allen MH, Fishman R (2011). Rapid acute treatment of agitation in individuals with schizophrenia: multicentre, randomised, placebo-controlled study of inhaled loxapine. Br J Psychiatry.

[11] European Medicines Agency. ADASUVE® Summary of product characteristics, 2014. http://www.ema.europa.eu/docs/en_GB/document_library/EPAR_-_Product_Information/human/002400/WC500139409.pdf. Accessed 21 July 2015.

[12] Rabinowitz JD, Wensley M, Lloyd P, Myers D, Shen W, Lu A (2004). Fast onset medications through thermally generated aerosols. J Pharmacol Exp Ther.

[13] Rabinowitz JD, Lloyd PM, Munzar P, Myers DJ, Cross S, Damani R (2006). Ultra-fast absorption of amorphous pure drug aerosols via deep lung inhalation. J Pharm Sci.

[14] Asmal L, Flegar SJ, Wang J, Rummel-Kluge C, Komossa K, Leucht S (2013). Quetiapine versus other atypical antipsychotics for schizophrenia. Cochrane Database Syst Rev.

[15] Komossa K, Rummel-Kluge C, Hunger H, Schmid F, Schwarz S, Duggan L (2010). Olanzapine versus other atypical antipsychotics for schizophrenia. Cochrane Database Syst Rev.

[16] Belgamwar RB, Fenton M (2005). Olanzapine IM or velotab for acutely disturbed/agitated people with suspected serious mental illnesses. Cochrane Database Syst Rev.

[17] Bellary S, Barnett AH (2006). Inhaled insulin (Exubera): Combining efficacy and convenience. Diab Vasc Dis Res.

[18] Fallowfield L, Atkins L, Catt S, Cox A, Coxon C, Langridge C (2006). Patients’ preference for administration of endocrine treatments by injection or tablets: results from a study of women with breast cancer. Ann Oncol.

[19] Liu G, Franssen E, Fitch MI, Warner E (1997). Patient preferences for oral versus intravenous palliative chemotherapy. J Clin Oncol.

[20] Atkinson MJ, Sinha A, Hass SL, Colman SS, Kumar RN, Brod M (2004). Validation of a general measure of treatment satisfaction, the Treatment Satisfaction Questionnaire for Medication (TSQM), using a national panel study of chronic disease. Health Qual Life Outcomes.

[21] Torrance GW, Furlong W, Feeny D (2002). Health utility estimation. Expert Rev Pharmacoecon Outcomes Res.

[22] Dolan P, Jones-Lee M (1997). The time trade-off: a note on the effect of lifetime reallocation of consumption and discounting. J Health Econ.

[23] Dolan P (1997). Modeling valuations for EuroQol health states. Med Care.

[24] Dolan P, Gudex C, Kind P, Williams A (1996). The time trade-off method: results from a general population study. Health Econ.

[25] Dolan P (1998). The measurement of individual utility and social welfare. J Health Econ.

[26] Robinson A, Dolan P, Williams A (1997). Valuing health status using VAS and TTO: what lies behind the numbers?. Soc Sci Med.

[27] Torrance GW, Feeny D (1989). Utilities and quality-adjusted life years. Int J Technol Assess Health Care.

[28] Torrance GW (1997). Preferences for health outcomes and cost-utility analysis. Am J Manag Care.

[29] Dolan P (1997). Aggregating health state valuations. J Health Serv Res Policy.

[30] World Health Organization. Life Tables by country. 2014. http://apps.who.int/gho/data/node.main.687?lang=en Accessed 15 July 2015.

[31] Battaglia J, Lindborg SR, Alaka K, Meehan K, Wright P (2003). Calming versus sedative effects of intramuscular olanzapine in agitated patients. Am J Emerg Med.

[32] Karagianis JL, Dawe IC, Thakur A, Bégin S, Raskin J, Roychowdhury SM (2001). Rapid tranquilization with olanzapine in acute psychosis: a case series. J Clin Psychiatry.

[33] Hsu WY, Huang SS, Lee BS, Chiu NY (2010). Comparison of intramuscular olanzapine, orally disintegrating olanzapine tablets, oral risperidone solution, and intramuscular haloperidol in the management of acute agitation in an acute care psychiatric ward in Taiwan. J Clin Psychopharmacol.

[34] Atkinson SE, Wilson PW (1995). Comparing mean efficiency and productivity scores from small samples: a bootstrap methodology. J Product Anal.

[35] Efron B (1981). Nonparametric estimates of standard error: the jackknife, the bootstrap and other methods. Biometrika.

[36] Briggs AH, Wonderling DE, Mooney CZ (1997). Pulling cost-effectiveness up by its bootstraps: a non-parametric approach to confidence interval estimation. Health Econ.

[37] Briggs A, Wild D, Lees M, Reaney M, Dursun S, Parry D (2008). Impact of schizophrenia and schizophrenia treatment-related adverse events on quality of life: direct utility elicitation. Health Qual Life Outcomes.

[38] Serper MR (2011). Aggression in schizophrenia. Schizophr Bull.

[39] Van Gelder MM, Bretveld RW, Roeleveld N (2010). Web-based questionnaires: the future in epidemiology?. Am J Epidemiol.

[40] Ekman A, Dickman PW, Klint A, Weiderpass E, Litton JE (2006). Feasibility of using web-based questionnaires in large population-based epidemiological studies. Eur J Epidemiol.

[41] Lund E, Gram IT (1998). Response rate according to title and length of questionnaire. Scand J Soc Med.

[42] Evans M, Holm Jensen H, Bøgelund M, Gundgaard J, Chubb B, Khunti K (2013). Flexible insulin dosing improves health-related quality-of-life (HRQoL): a time trade-off survey. J Med Econ.

[43] Ridderstråle M, Evans LM, Jensen HH, Bøgelund M, Jensen MM, Ericsson Å, Jendle J (2016). Estimating the impact of changes in HbA1c, body weight and insulin injection regimen on health related quality-of-life: a time trade off study. Health Qual Life Outcomes.

